# Regulation of inflammation in Japanese encephalitis

**DOI:** 10.1186/s12974-017-0931-5

**Published:** 2017-08-14

**Authors:** Nils Lannes, Artur Summerfield, Luis Filgueira

**Affiliations:** 1 Unit of Anatomy, Department of Medicine, University of Fribourg, Route Albert-Gockel 1, Fribourg, Switzerland; 2 Institute of Virology and Immunology, Sensemattstrasse 293, Mittelhäusern, Switzerland; 3 Department of Infectious Diseases and Pathobiology, Vetsuisse Faculty, University of Bern, Langassstrasse 122, Bern, Switzerland

## Abstract

**Background:**

Uncontrolled inflammatory response of the central nervous system is a hallmark of severe Japanese encephalitis (JE). Although inflammation is necessary to mount an efficient immune response against virus infections, exacerbated inflammatory response is often detrimental. In this context, cells of the monocytic lineage appear to be important forces driving JE pathogenesis.

**Main body:**

Brain-infiltrating monocytes, macrophages and microglia play a major role in central nervous system (CNS) inflammation during JE. Moreover, the role of inflammatory monocytes in viral neuroinvasion during JE and mechanisms of cell entry into the CNS remains unclear. The identification of cellular and molecular actors in JE inflammatory responses may help to understand the mechanisms behind excessive inflammation and to develop therapeutics to treat JE patients. This review addresses the current knowledge about mechanisms of virus neuroinvasion, neuroinflammation and therapeutics critical for JE outcome.

**Conclusion:**

Understanding the regulation of inflammation in JE is challenging. Elucidation of the remaining open questions will help to the development of therapeutic approaches avoiding detrimental inflammatory responses in JE.

## Background

Japanese encephalitis (JE) is an acute and uncontrolled inflammatory disease of the central nervous system (CNS) in humans, especially affecting children. JE is caused by Japanese encephalitis virus (JEV), a neurotropic single-stranded RNA virus belonging to the *Flaviridae* family, *Flavivirus* genus. JEV is transmitted by mosquito vectors in a zoonotic cycle involving wild aquatic bird reservoirs and pigs as amplifying hosts. Humans are dead-end hosts, because low viremia does not allow further viral spread [1]. The incubation period is 5–15 days and common symptoms include fever, headache, vomiting and neurologic symptoms such as paralysis and movement disorders. Seizures can occur in severe cases [1–3]. However, less than 1% of JEV infections are symptomatic. JE has an estimated incidence of 70,000 human cases annually, including 5–30% fatal cases and 30–50% of survivors showing permanent neurological sequelae [4, 5]. Therefore, JEV is a leading cause of severe encephalitis in Asia where it is epidemic in northern regions and endemic in southern regions [6]. JEV-infected areas extend from Pakistan to Japan and from Korea to Indonesia [5, 6]. In 1995, Eastern Pacific regions and Northern Australia became infected [7]. During the 2000s, JEV RNA, but no infectious virus, has occasionally been detected in Italy [8, 9]. Recently, JEV RNA was detected in Angola during a yellow fever outbreak in 2016 [10]. Moreover, JEV distribution exists under both tropical/subtropical and temperate climates [11]. Altogether, JEV’s spread to new regions represents an increasing risk [9, 12] and JEV may become a worldwide public health concern.

JEV-induced inflammation contributes to disease severity by inducing neuronal cell death [13, 14], inhibiting the proliferation and differentiation of neural progenitors [15] and disrupting the blood-brain barrier (BBB) [16, 17]. Importantly, JEV-induced inflammation further modulates cytokine/chemokine production [18] as well as the activation and migration of cells [13, 19]. Therefore, production of soluble factors and trafficking of immune cells may lead towards either disease progression or recovery through promotion of protective immune responses.

Although vaccination programs for travellers and inhabitants of regions at risk contribute to prevention of JE [5], unvaccinated individuals remain at risk. Nowadays, no specific cure exists for individuals developing JE [2, 3]. Therefore, the development of an effective and specific curative treatment for JE patients is necessary and requires further investigations.

The present review aims to understand cellular and molecular mechanisms involved in inflammatory responses to JEV infection and to identify key regulators of inflammation in JE. The understanding of these mechanisms may be central for the development of specific curative treatment of JE. Accordingly, the present review presents potential pharmaceutical candidates with promising anti-inflammatory or anti-viral properties.

## Tropism and kinetics of viral replication

JEV is detected in various anatomical sites and propagates in various cell types including non-immune and immune cells (Table 1). Individuals are infected by JEV upon the bite of an infected mosquito. The dermis is supposedly the primary site of infection and JEV may propagate in cells of the dermal tissue before reaching lymphoid organs, probably transported by infected migratory immune cells such as Langerhans cells [3, 20, 21]. As a result, JEV has been detected and isolated from the spleen and lymph nodes of infected patients [16, 22–24]. During the acute phase of human JE, the virus can be isolated from blood cells [25, 26]. This can be associated with a low and transient viremia [2], and was also described in mouse models [16, 22, 27]. JEV may also replicate in human lymphocytes, albeit at low rate and possibly depending on the virus strain [28, 29]. Monocytes allow viral propagation in vitro by JEV without evidence of cell death [28, 30]. Interestingly, immature but not mature human monocyte-derived dendritic cells (DC) are susceptible to JEV infection in vitro resulting in virus propagation [31, 32]. It would still be necessary to quantify viral RNA over time in mature human DC in order to evaluate virus replication. Also murine DC allows virus replication in vivo and in vitro [33–36], but not the release of infectious viral particles in vitro [34]. Thus, the identification of the causes for the unproductive JEV infection of murine DC and mature human DC would help to understand key regulators for viral propagation in the latter cell types and others. Moreover, murine plasmacytoid DC (pDC) are permissive to JEV [35]. Human [37] and murine [29, 34–36] macrophages support virus propagation in vitro. But, JEV is cytotoxic to macrophages [38].

**Table 1:** Cellular source of viral propagation with body localization and abilities of neuroinvasion

Body localisation	Cell type	JEV propagation	CNS infiltration
Blood	Granulocytes	−	+
	Immature/mature DC	+/−	n.i.
	pDC	+	n.i.
	Monocyte	+	+
BBB	Endothelial cell	+	
	Pericyte	+	
	Astrocyte	+	
CNS	Microglia	+	
	Neurons	+	
Tissues	Macrophage	+	+

Tissue include the brain; *n.i*. no information

The mechanism behind JEV entry into the CNS is not well understood. Nevertheless in mice, the BBB disrupts only after viral neuroinvasion [16] indicating that neural infection is not necessary a consequence of the breakdown of the BBB, but the other way around. Interestingly, JEV productively infects rodent microvascular brain endothelial cells [39] that may be functionally affected in terms of their role for the BBB [21, 39, 40]. As these form the blood-brain barrier, this may represent a possible way of JEV transmission to brain tissue cells [41], after which the virus could infect pericytes [42] and astrocytes [39, 43]. Also the microglia cells representing CNS-resident macrophages are susceptible to JEV infection in vivo [44]. Murine microglia is also productively infected by JEV for up to 16 weeks in vitro [45]. Although human microglia cells allow JEV replication, infectious virus is transmitted to susceptible cells in a cell-cell dependent manner [46].

After entering into the brain, JEV is found in the nervous tissue and cerebrospinal fluid (CSF) in JE patients [26, 47–49] and in the mouse model [16, 23, 24, 49]. In the human brain, JEV antigen is mainly detected in the nuclear grey matter, including the thalamus, the hypothalamus, the hippocampus and the substantia nigra [48–50], where most of brain lesions appear [1, 51]. Also in macaques intranasally infected with JEV, viral antigen is detected in the thalamic and brain stem nuclei [44]. Neuronal cells are reported to be the most important target cells of JEV [16, 44, 52], allowing virus propagation [49]. Rodent models indicate that JEV has a particularly high tropism for neuronal precursors and developing neurons, affecting their proliferation and development [15, 52, 53]. Furthermore, JEV infection can cause direct neuronal damage [28, 52], which is further enhanced by JEV-induced inflammation [54, 55]. Actually, some studies suggested a correlation between the fatal outcome and the degree of inflammatory responses, although this remains controversial [3, 44, 49, 50, 56–58].

As a potent model for the study of human disease, pigs infected with JEV present a high viral loads in secondary lymphoid tissue. However, these animals have high viremia without association of JEV to blood leukocytes [59]. Also, JEV is consistently found in CNS tissues of animals, even if they did not show clinical symptoms [60]. Unlike human and murine cells, both porcine monocytes-derived DC and macrophages efficiently support virus replication (unpublished observations, A. Summerfield).

## Inflammation in JE

### Inflammatory cells in JE

Upon JEV infection, various immune cell types increase in number in various compartments of the periphery, including the spleen, lymph nodes and blood. In mice, numbers of macrophages, inflammatory monocytes, granulocytes and pDC increase in lymphoid tissues, such as spleen and lymph nodes [19, 29, 36]. Although not dramatically, natural killer cell population decreased in the spleen of JEV-infected mice [19]. In the blood, leucocytosis characterized by high numbers of monocytes and neutrophils was found in human patients [50, 61]. Similarly, JEV-infected mice show an increase of monocytes and neutrophils in blood [17, 19].

During JE, various peripheral immune cell types infiltrate the CNS (Table 1). In JE, infection of microvascular endothelial cells enhances the expression of adhesion molecules leading to transmigration of leukocytes [39]. In macaques intranasally infected with JEV, evidence of endothelial cells activation is also found [44]. However, DC is critical to maintain the integrity of the BBB by modulating the expression of tight junction and adhesion molecules in mice intraperitoneally infected with JEV [62] and provided adequate signal to differentiate/activate monocytes regulating neuroinflammation and viral propagation into the brain [23]. In the CSF of JE patients, leukocytes count of polymorphonuclear and mononuclear cells increases [15, 63]. In human brains of lethal JE cases, detection of perivascular erythrocytes and peripheral inflammatory mononuclear cells infiltrates indicates major vascular damage [49]. Also, perivascular infiltrates and multifocal lymphohistiocytic meningitis are a hallmark of JEV-infected pigs [59] and macaques [44]. In mice, macrophages/monocytes are the majority of brain-infiltrating inflammatory myeloid cells [19, 24, 49, 64, 65]. In addition, granulocytes and NK cells also infiltrate the brain of JEV-infected mice [19, 65, 66].

In addition to the contribution of peripheral immune cells infiltrating the brain, brain-resident cells interact with JEV upon infection of the brain. Activated microglial cell nodules develop, and the number of reactive astrocytes increases in human [49], macaques [44] and mouse [67]. Such glial nodules and evidences of neuronal degeneration and necrosis were also found [44, 59]. Microglia has been proposed to play a major role in neuronal cell death through release of pro-inflammatory mediators [13].

### Inflammatory and anti-viral mediators in JE

Inflammation is a hallmark of JE with various inflammatory chemokines and cytokines having potential anti-viral activity in different body localizations. Inflammatory immune cells described previously, in addition to non-immune cells, can be sources of those mediators in response to JEV (Table 2). Human DC produce the cytokines tumour necrosis factor (TNF)-α, interleukin (IL)-6, type-I interferons (IFN) and the chemokines CCL2, CCL5, CXCL8 (IL-8) and CXCL10 in response to JEV [31, 32]. Upon exposure to JEV, murine DC produce TNF-α IL-6, IL-12, type-I IFN and CCL2 [33–35]. However, contrasting results of the various studies may be virus strain-specific since Beijing-1 strain induced TNF-α in murine DC [34, 35] whereas JEV P3 strain did not [33]. Additionally, murine pDC produce type-I IFN in response to JEV [35]. Human [24, 37] and murine [24, 34, 35, 38, 55] macrophages produce TNF-α, IL-6, IFN-α and CCL2 in response to JEV in vitro. Moreover, CXCL-8 has been measured from human macrophages [37] and IL-12, IFN-β and IFN-γ from murine macrophages [34, 35, 38, 55]. Both human [46] and rodent [24, 54, 68] microglia produce CCL2 upon JEV exposure. In addition, human microglia produce CXCL9 and CXCL10 [46]. Rodent microglia also release cytokines such as TNF-α, IL-1β, IL-6 [13, 24, 68, 69] and the chemokine CCL5 [13, 70]. Upon intracranial infection of mice, microglia stain for TNF-α, IL-1β, IL-6 and IL-18 [14, 24] and brain infiltrating monocytes for TNF-α and IL-6 [24]. In JEV-macaques, microglia stains for TNF-α [44]. As part of the BBB, human endothelial cells produce TNF-α and IFN-β in response to JEV [71] and rat endothelial cells produce CCL5 [39]. Rodent pericytes produce IL-6 upon JEV treatment [42]. Astrocytes of rodent origin release the cytokines IL-1β, IL-6, IL-18 and the chemokine CCL5 [13, 14, 43, 70]. Moreover, both JEV-infected human and murine astrocytes are responsible for the production of CXCL10 [72]. Brain sections of JEV-infected macaques reveal staining for TNF-α and IFN-α in astrocytes [44]. Finally, neurons of murine origin release TNF-α, IL-6, IL-12, IFN-α, IFN-γ and CCL2 in response to JEV treatment [73–75]. In JEV-infected macaques, neuronal cells are positive for IFN-α staining [44].

**Table 2:** Cellular source of cytokines and chemokines

	TNF-α	IL-1β	IL-6	IL-12	IL-18	IFN-α/β	IFN-γ	CCL2	CCL5	CXCL8	CXCL9	CXCL10
Granulocytes												
DC	+		+	+		+		+	+	+		+
pDC						+						
Monocyte	+		+									
Endothelial cell	+					+			+			
Pericyte			+									
Astrocyte	+	+	+		+	+			+			+
Microglia	+	+	+		+			+	+		+	+
Neurons	+		+	+		+	+	+				
Macrophage	+		+	+		+	+	+	+	+		

#### Chemokines

Chemokine axis plays a crucial role in JE pathogenesis by attracting migrating cells of the lymphatic and blood circulation systems. In JEV-infected mice, the spleen presents upregulated levels of CCL2 and CXCL10 [64, 76]. Alongside, serum of JEV-infected mice has higher levels of CCL2, CCL4 and CXCL10 as compared to uninfected animals [16, 24]. In JE-human patients, plasma and serum contain enhanced levels of CXCL8 and CCL5 [63, 77]. In response to a vaccine containing a live attenuated strain of JEV, serum of immunized human subjects increase levels of IL-8, CCL2, CCL3 and CCL4 [78]. At the CNS level, CSF of JEV-infected mice presents elevated levels of CCL2 [24]. In the CSF of JE-patients, IL-8 and CCL5 are found in elevated levels [63, 77, 79]. Brains of JEV-infected mice have increased levels of CCL2, CCL3, CCL4, CCL5 and CXCL10 [15, 16, 36, 54, 64, 68, 76]. Interestingly, cortex, striatum, thalamus, hippocampus, sub-ventricular zone and midbrain are found to express high levels of CCL2, with highest levels found in the cortex [15, 36, 80]. Moreover, enhanced mRNA levels of CCR1, CCR2, CCR4, CCR5, CXCR2 and CXCR3 have been measured in the brain of JEV-infected mice [19, 67].

The CCL5-CCR5 axis is involved in recovery and may control the level of inflammation during JE. In JE patients, higher levels of CCL5 have been found in CSF of non-survivors than in survivors [77]. Although neutralization of CCL5 does not affect the adhesion of peripheral blood mononuclear cells and neutrophils on a monolayer of human endothelial cells, transmigration of leukocytes across the monolayer is inhibited [39]. In vitro neutralization of CCL5, produced by JEV-infected murine glial cells, inhibits the attraction of murine monocytes/macrophages [18, 70]. However, CCR5-knock-out (KO) mice are more susceptible to lethal JEV infection upon both intravenous [65] and intraperitoneal injection [81] although only intravenous infection leads to higher viral burden in the brain and spinal cord compared to control animals [65]. CCR5-KO mice also present increased numbers of brain infiltrating monocytes and granulocytes, as well as activated microglia and macrophages. Moreover, CCR5-KO mice show higher levels of the mediators IL-1β, IL6, CCL2, CCL3, CCL4 and CCL5 [81].

In the process of brain-invasion by inflammatory cells and potential viral neuroinvasion during JE, the CCL2-CCR2 axis plays a crucial role which further affect brain inflammatory environment. Upon intradermal infection of mice with JEV, CCL2 deficiency increases mortality and morbidity of animals which presented higher viral loads in brain and spinal cord in comparison to control animals [19]. Although neutralization of CCL2 produced by JEV-infected murine glia reduces attraction of murine monocytes/macrophages cell line in vitro [70], monocytes and granulocytes accumulate in the brain of JEV-infected CCL2-deficient mice [19]. Moreover, higher expression levels of the chemokine ligands CCL3, CCL4, CCL5 and CXCL9, as well as the receptors CCR1, CCR2, CCR4 and CCR5 are detected in the brain of the these animals [19]. Interestingly, CCR2 deficiency in mice leads to decreased susceptibility against lethal infection by JEV, but with no difference in viral load in the brain. Moreover, CCR2 deficiency results in a reduced accumulation of monocytes, but not granulocytes in the brain of JEV-infected mice [19]. However, in another mouse model where DC were ablated, CCR2 deficiency increases the speed of accumulation of monocytes into the CNS compared to control animals [23] indicating that the speed of monocytes into the CNS is CCR2-dependent. Finally, JEV-infected CCR2-deficient mice have also reduced expression of CCL3, CCL4, CCL5 and CCR1 in the brain [19].

#### Cytokines

Cytokines are essential to mount a potent immune response against JEV providing danger signals and anti-viral activity. In JEV-infected mice, increased levels of IL-12 and IFN-γ are found in lymph nodes and spleen [28, 64]. Serum of JEV-infected mice contains TNF-α, IL6, IL-18, as well as IFNs [16, 24, 27, 35, 36, 82]. In JE-human patients, increased levels of TNF-α [83] and IFN-α [84] are measured in blood. At the CNS level, CSF of mice shows elevated levels of TNF-α, IL6 and IL-18 [24] upon JEV infection. In the brain tissue of JEV-infected mice, upregulated levels of TNF-α, IL-1β, IL-6, IL-12, IL-18, IFN-γ and CXCL10 have been reported [14–16, 36, 54, 64, 68, 72, 82]. Notably, the cerebral cortex presents the highest level of TNF-α, IL-6 and types I and II IFNs [15, 36, 54, 80]. In JE-patients, an increase of TNF-α, IL-6 and IFN-α is detected in the CSF [77, 79, 83, 84].

In JE, TNF-α has a major impact on the dynamic of inflammation and the outcome of the disease. For instance, high levels of TNF-α in serum and CSF of patient is associated with lethality [83]. Intracranial administration of silencing RNA (siRNA) against the TNF receptor-associated death domain (TRADD) decreases mortality of intravenously JEV-infected mice [85], reduces virus neuroinvasion and neuronal cell death [67]. In vitro neutralization of TNF-α derived from JEV-infected murine microglia cultures also reduces cytotoxicity to neuronal cells [13]. Interestingly, the brain of JEV-infected mice treated with TRADD siRNA has an abrogated expression of adhesion molecules and lower levels of brain-infiltrating neutrophils were found [67]. In vitro neutralization of TNF-α inhibits the production of CCL5 from JEV-infected glia. As a result, the latter supernatants show reduced chemotactic activity towards murine macrophages [18]. Furthermore, TNF-α enhances the production of CCL5 by uninfected murine astrocytes [18] which may enhance the recruitment of leukocytes in JE. Finally, TRADD siRNA treatment of JEV-infected mice reduces the expansion of astrocytes and the activation of microglia. Brain of these mice have reduced levels of the mediators TNF-α, IL-6, IL-12, IFN-γ and CCL2, as well as the receptors CCR1, CCR2 and CXCR3 [67].

## Molecular components of JEV recognition

### Pattern recognition receptors in the recognition of JEV

JEV has been found to interact with the toll-like receptor 2 (TLR2) in neurons and TLR3 and/or TLR7 in microglial cells [75, 86, 87]. Upon JEV infection, KO of TLR3 in mice enhances lethality and severity of JE, as wells as viral loads in the spinal cord and the brain in comparison to control animals [36]. Actually, knocking-down (KD) of TLR3 with small hairpin RNA increases viral load in murine microglia [87]. Additionally, TLR3-KO mice present stronger permeability of the BBB and increased brain-infiltration of inflammatory monocytes with activation of macrophages/microglia. These animals also show higher levels of systemic IL-6 and IFN-β. In the brain and the spinal cord, higher mRNA levels of IL-6, type-I IFN, CCL2, CCL5 and CXCL10 are detected, whereas CCL3 and CCL4 are only found in the spinal cord [36]. KD of TLR3 in murine microglia reduces the secretion of TNF-α [87]. In contrast to TLR3-KO mice, JEV-infected TLR4-KO mice show reduced severity and lethality of JE and lower viral loads are detected in the brain than in wild-type (WT) animals. Interestingly, TLR4-KO mice do not show any difference in brain-infiltration of inflammatory monocytes and activation of macrophages/microglia. However, these animals secrete high levels of systemic IFN-β [36]. Otherwise, subcutaneous JEV infection of systemic TLR7-KD mice leads to increased mortality in comparison with control animals, whereas specific KD of brain TLR7 does not influence the mortality of the animals. Interestingly, systemic TLR7-KD mice show higher brain viral loads than brain TLR7-KD in mice, indicating the importance of peripheral virus detection in order to control JEV neuroinvasion. Moreover, systemic TRL7-KD mice secrete higher levels of brain IL-6 than brain TLR7-KD in mice. Nevertheless, KD of TLR7 leads to stronger brain-infiltration of monocytes and neutrophils, stronger activation of microglia and higher levels of TNF-α, IL-6 and CCL2, but lower levels of IFN-α in the brain of both models [75]. At the cytoplasmic level, the melanoma differentiation-associated protein 5 (MDA5) and retinoic acid-inducible gene 1 (RIG-I) are important [86]. KD of RIG-I increases viral load in murine microglia [87]. In addition, blockade of RIG-I decreases the release of TNF-α, IL-6 and CCL2 from murine microglia [87] and neurons [73] and of IL-12 from neurons [73]. Overall, TLR3 and RIG-I may rather be protective. Furthermore, TLR7 seems to initiate protective inflammatory signals against JE. In contrast, TLR4 may contribute to pathological JE. JEV has also been reported to interact with the C-type lectin domain family 5 member A (CLEC5A) receptor leading to the phosphorylation of the DNAX activation protein of 12 kDa in human and murine macrophages [24]. In mice infected intraperitoneally with JEV, administration of anti-CLEC5A antibodies via the same route diminishes the susceptibility to lethal JEV infection and reduces JEV neuroinvasion. These animals maintain the integrity of the BBB and reduce brain-infiltration of inflammatory myeloid cells and proliferation of macrophages/microglia. Moreover, lower levels of TNF-α, IL-6, IL-18 and CCL2 are found in serum and CSF [24]. Additionally, KD of NOD-like receptor family pyrin domain containing 3 (NLRP3) by siRNA decreases the production of IL-1β and IL-18 in murine microglia upon JEV treatment. Nevertheless, activation of NLRP3 in JEV-treated microglia is due to secondary signals such as the activity of caspase-1, itself influenced by reactive oxygen species [68]. Thus, CLEC5A and NLRP3, both associated with the inflammasome activation, seem to have major contribution to pathological JE.

### Downstream signalling pathways upon JEV recognition

Upon JEV recognition, downstream signalling pathways are induced and may involve adaptor proteins such as myeloid differentiation primary response gene 88 (MyD88) [34, 86]. Upon JEV exposure in vitro, both DC and macrophages from MyD88-KO mice reduce the production of IL-6 and IL-12 in comparison to cells from WT-mice. Moreover, macrophages of MyD88-KO animals also release less TNF-α [34].

Furthermore, kinases such as protein kinase B (Akt) [38], phosphoinositide 3-kinase (PI3K) [32, 38], p38 mitogen-activated protein kinase (p38MAPK) [32, 34, 38, 55, 73, 87] and signal-regulated kinase (ERK) [39, 70, 87–89] are found to be involved in signalling pathways upon JEV recognition. Upon inhibition of p38MAPK or PI3K, human DC produces less TNF-α, type-I IFN and CXCL8 [32]. Murine DC diminishes the production of TNF-α, IL-6 and IL-12 after inhibition of p38MAPK [34]. In murine macrophages, inhibition of p38 MAPK reduce the production of TNF-α, IL-6, IL-12, IFN-γ and CCL2, resulting in the loss of cytotoxicity to neuronal cells [55]. In murine microglia, inhibition of p38MAPK or ERK leads to reduced production of TNF-α, IL-6 and CCL2 [87]. Inhibition of ERK also leads to abrogated production of TNF-α and IL-1β by rat microglia [88]. Finally, inhibition of ERK leads to reduced release of CCL5 glial cells [70] and endothelial cells [39] of rodents. In addition, inhibition of ERK leads to reduced expression adhesion molecules in rodent endothelial cells [39].

Finally, transcription factors such as interferon regulated factor 3 (IRF3)/IRF7 [36, 75, 86, 90–92], activator protein 1 [87] and nuclear factor kappa-light-chain-enhancer of activated B cell (NF-κB) [38, 39, 70, 73, 75, 87] are found to be implicated during JEV infection. In rat microglia, inhibition of NF-κB abrogates the production of TNF-α and IL-1β by rat microglia [88]. In rodent endothelial cells, inhibition of NF-κB reduces the production of CCL5 and adhesion molecules [39].

Importantly, the activity of inflammatory components have also been described to depend on the janus kinase-signal transducer and activator of transcription (JAK-STAT) signalling pathway in JEV infection. This requires the activation of STAT1 [24, 36, 92] and the expression of IFN-dependent and IFN-independent IFN-stimulated genes [36, 37].

### MicroRNA upon JEV treatment

MicroRNAs (miRNA) contribute to the regulation of gene expression in various cell types including astrocyte, microglia and neuronal cells upon JEV infection. Replicative JEV modulates cellular miRNA expressions in time- and dose-dependent manners [93–96]. JEV-influenced miRNAs target elements of pathogen recognition [95, 96] and downstream signalling pathways [93, 94, 97, 98], as well as the JAK-STAT signalling pathway [95, 99–102].

JEV may modulate the expression of miRNA resulting in inhibition of inflammatory responses. JEV downregulates the expression of miR-432 reducing the production of TNF-α and IL-6 and suppressed JEV replication in human microglia [101]. Moreover, JEV upregulates miR-146a expression reducing the expression of TNF-α and IL-6 in both human and murine microglia [99, 100] and of IL-1β, IFN-α and IFN-β in murine cells [100]. However, miR-146a enhances JEV replication in human microglia [99]. Finally, JEV upregulates miR-301a expression, which inhibits type-I IFN production in human and murine neuronal cells. However, miR-301a promotes JEV replication [102].

Otherwise, the influence of JEV on the expression of miRNA may result in enhanced inflammatory responses as well as increased JE lethality and severity. Such miRNAs represent potential therapeutic targets. In murine microglia cells, JEV upregulates miR-29b expression inducing microglia activation and increased expression of TNF-α, IL-1β, IL-6 and CCL2. Inhibition of miR-29b reduces the expression of inflammatory mediators [98]. In human glioblastoma cells, JEV downregulates the expression of miR-370 enhancing expression IFN-β, inhibited by using a miR-370 mimic. Virus replication rate and JEV-induced cell injury are also inhibited by using miR-370 mimic, but restored by further inhibition of the miR-370 mimic activity [97]. In both human astrocytoma cells and murine microglia cells, JEV upregulates miR-19b-3p [93] and miR-15b [96] increasing the production of TNF-α, IL-1β, IL-6 and CCL5 [93, 96], as well as IL-12, IFN-β and CCL2 [96]. Inhibition of miR-19b-3p or miR-15b suppresses the production of these inflammatory mediators. Importantly, intravenous administration of miR-19b-3p or miR-15b antagonist reduces neuroinflammation and lethality of mice upon intracranial infection with JEV [93, 96]. Finally, JEV upregulates the expression of miR-155 in both human and mouse brain tissue [94]. miR-155 expression enhances the production of TNF-α, IL-6, IFN-β and CCL2 in murine microglia cells [94], but reduces the expression of TNF-α, IL-1β and IFN-β in human microglia cells [103]. miR-155 also suppresses JEV replication in human microglia [103]. Nevertheless, intravenous administration of anti-miR-155 attenuates neuroinflammation, microglial activation and mortality in intravenously JEV-infected mice [94].

## Therapeutic candidates to control JE

There is still no specific curative treatment for JE. Nevertheless, anti-inflammatory therapeutic candidates are currently under evaluation. However, such candidates must have specific anti-viral effects against JEV. The following drugs have shown promising effects during the course of a JEV infection.

Arctigenin is a polyphenolic lignan compound found in plants of the Asteraceae family. Upon JEV infection, arctigenin inhibits the activation of kinases such as p38-MAPK, ERK and Akt abrogating microglial activation and production of cytokines such as TNF-α, IL-6, IFN-γ and CCL2 [104]. Arctigenin also reduces JEV-induced neuronal cell death [104]. Importantly, arctigenin reduces brain tissue viral load, induces neuroprotection and protects from JE lethality [104].

Minocycline is a semi-synthetic tetracycline antibiotic. Upon JEV infection, minocycline reduces the phosphorylation of kinases such as PI3K, Akt and p38 MAPK, as well as the transcription factor NF-κB [38]. As a result, minocycline reduces the production of TNF-α, IL-6, IL-12, IFN-γ and CCL2 in the brain [64, 105] and inhibits microglial activation [15, 105]. Interestingly, minocycline also limits the infiltration of innate immune cells into the brain of JEV-infected mice [15, 64]. Finally, minocycline reduces viral replication and the expression of viral antigen in the brain and confers complete protection against JE [64, 105].

2-(2-Methyl-quinoline-4ylamino)-N-(2-chlorophenyl)-acetamide (PP2) is a synthetic anilidoquinoline derivative. In microglia and neuron/glia cultures infected with JEV, PP2 suppresses the activation of NF-κB decreasing the production of TNF-α, IL-1β and CCL5 [89, 106]. PP2 also reduces neurotoxicity of JEV [89, 106, 107]. Although PP2 A does not affect JEV replication, it reduces the phosphorylation of the viral protein NS3 by phospotyrosine resulting in a diminished release of infectious virus particles from neuron/glia cultures [89, 106]. PP2 reduces mature intracellular and brain viral load and confers neuroprotection [107]. Importantly, Mice treated with PP2 show complete protection from lethal JEV infection [107].

Vivo-morpholinos (MOs) are synthetic uncharged anti-sense oligomer analogs of DNA or RNA targeting specific genomic region. MOs targeting JEV genome reduces phosphorylation of the kinases p38 MAPK and ERK as well as the transcription factor NF-κB. Consequently, MOs inhibit the production of TNF-α, IL-6, IFN-γ and CCL2 in the brain and microglial activation [108]. MOs abrogate neurodegeneration, reduce viral load in the brain and protects from JE [108].

In conclusion, therapeutic candidates have anti-inflammatory, antioxidant and JEV-specific anti-viral activities. Importantly, these molecules enter the brain even though administration is in the periphery. Nevertheless, none of these drugs has yet been approved for the treatment of JEV infection in humans.

## Conclusions

**Fig. 1:**
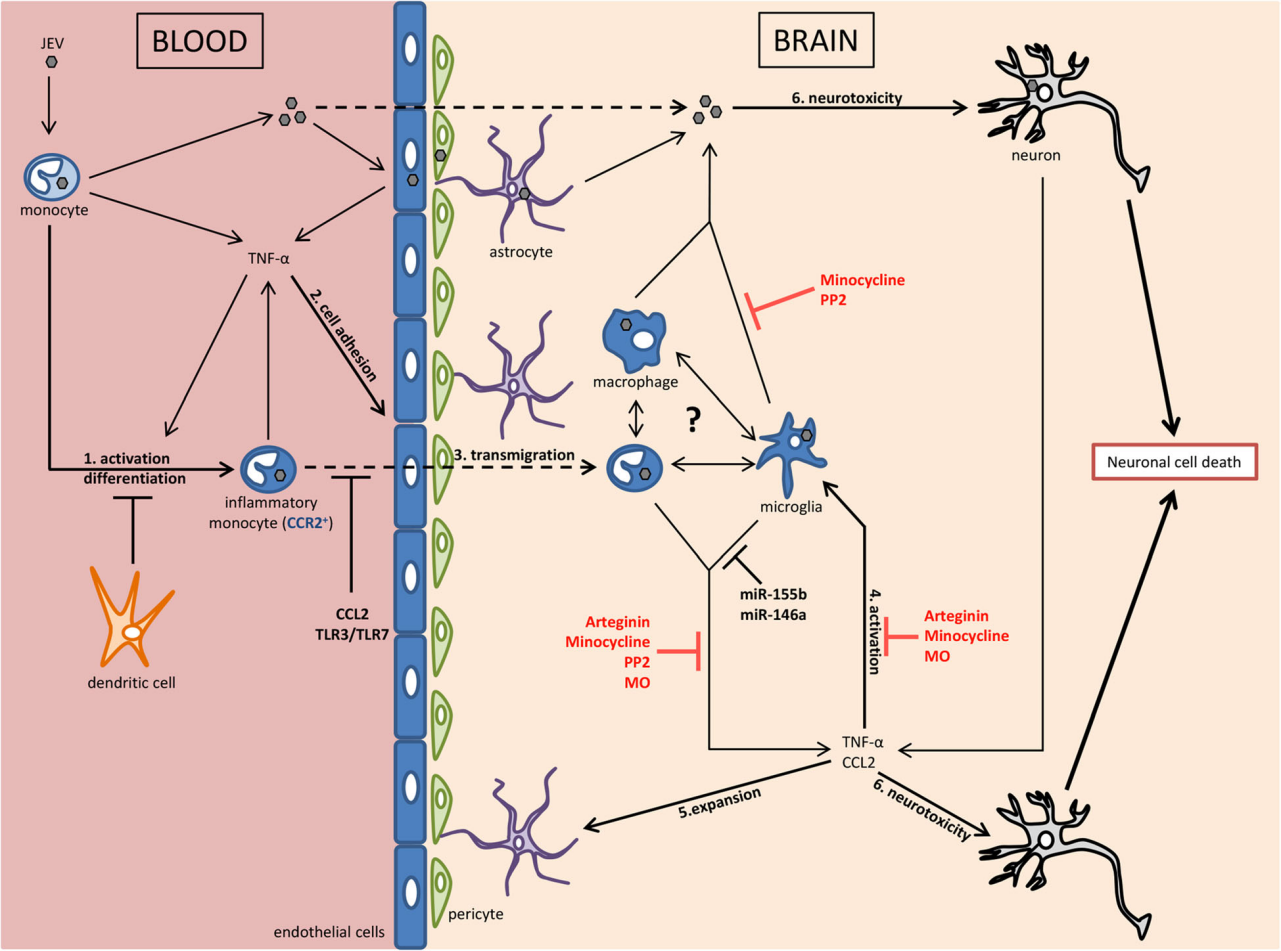
Monocytes participate in inflammation and viral propagation and produce TNF-α upon JEV infection. (*1*) TNF-α is implicated in the activation and differentiation of monocytes as well as (*2*) the expression of adhesion molecules on endothelial cell surface constituting the BBB which participates in (*3*) the transmigration of inflammatory monocytes through the BBB. (*4*) In the brain, inflammatory monocytes, macrophages, microglia and neuronal cells contribute to viral propagation and neuroinflammation. (*5*) TNF-α and CCL2 lead to microglial activation and astrocytic expansion. (*6*) Ultimately, neuronal cell death results of direct cytotoxicity of JEV and indirect effects of inflammatory mediators. (in *black*) Chemokines (CCL2) and pattern recognition receptors (TLR3, TLR7) inhibit cellular and virus neuroinvasion and miRNAs (miR-155b, miR-146a) suppress microglia-derived inflammatory responses during JE. (in *red*) Promising therapeutic candidates inhibit neuroinflammation in JE

Systemic and neural inflammation contributes to the anti-viral immune response, but is also responsible for the brain pathology in JE. The balance between anti-viral and brain damaging inflammatory effects is probably the key predictor of the outcome. In that respect, various cells and factors contribute to that balance but may also contribute to dysregulation and pathology. Cells of the monocytic lineage appear to play a central role in inflammatory responses and pathogenesis in JE (Fig. 1). In particular, TNF-α and the axis CCL2-CCR2 has a major impact in neuroinvasion of immune cells including inflammatory monocytes. The activation of TLR3/TLR7 signalling pathways, the activity of CCL2 as well as the intervention of DC inhibits peripheral inflammatory responses to JEV. Furthermore, miR-155b and miR-146a suppress brain inflammation. Finally, therapeutic candidates such as minocycline and PP2 present anti-inflammatory and anti-viral activity upon JEV infection.

Since all therapeutic candidates inhibit microglia activation which are a main producer of inflammatory mediators, future directions of the development of therapeutics should take care of the targeting microglial activation in order to reduce JE neuroinflammation.

## Abbreviations

Akt: Protein kinase B
BBB: Blood-brain barrier
CLEC5A: C-type lectin domain family 5 member A
CNS: Central nervous system
CSF: Cerebrospinal fluid
DC: Dendritic cell
ERK: Signal-regulated kinase
IFN: Interferon
IL: Interleukin
JE: Japanese encephalitis
JEV: Japanese encephalitis virus
KD: Knock-down
KO: Knock-out
MDA5: Melanoma differentiation-associated protein 5
miRNA: Micro RNA
MO: Vivo-morpholinos
NF-κB: Nuclear factor kappa-light-chain-enhancer of activated B cell
NLRP3: NOD-like receptor family pyrin domain containing 3
p38MAPK: p38 mitogen-activated protein kinase
pDC: plasmacytoid dendritic cell
PP2: 2(2-methyle-quinoline-4ylamino)-N-(2-chlorophenyl)-acetamide
RIG-I: Retinoic acid-inducible gene 1
siRNA: Silencing RNA
TLR: Toll-like receptor
TNF: Tumour necrosis factor
TRADD: TNF receptor-associated death domain
WT: Wild-type
